# Influence of cerebral microbleeds on mechanical thrombectomy outcomes

**DOI:** 10.1038/s41598-022-07432-9

**Published:** 2022-03-07

**Authors:** Seong-Joon Lee, Yang-Ha Hwang, Ji Man Hong, Jin Wook Choi, Ji Hyun Park, Bumhee Park, Dong-Hun Kang, Yong-Won Kim, Yong-Sun Kim, Jeong-Ho Hong, Joonsang Yoo, Chang-Hyun Kim, Sung-Il Sohn, Jin Soo Lee

**Affiliations:** 1 Department of Neurology, Ajou University School of Medicine, Ajou University Medical Center, 164, World cup-ro, Yeongtong-gu, Suwon, Gyeonggi-do 16499 Republic of Korea; 2 Department of Neurology, School of Medicine, Kyungpook National University, Daegu, Republic of Korea; 3 Department of Radiology, Ajou University School of Medicine, Ajou University Medical Center, Suwon, Republic of Korea; 4 Office of Biostatistics, Ajou Research Institute for Innovative Medicine, Ajou University Medical Center, Suwon, Republic of Korea; 5 Department of Biomedical Informatics, Ajou University School of Medicine, Suwon, Republic of Korea; 6 Department of Neurosurgery, School of Medicine, Kyungpook National University, Daegu, Republic of Korea; 7 Department of Radiology, School of Medicine, Kyungpook National University, Daegu, Republic of Korea; 8 Department of Neurology, Keimyung University Dongsan Medical Center, Daegu, Republic of Korea; 9 Department of Neurology, Yonsei University College of Medicine, Yongin Severance Hospital, Yongin, Republic of Korea; 10 Department of Neurosurgery, Keimyung University Dongsan Medical Center, Daegu, Republic of Korea

**Keywords:** Biomarkers, Diseases, Neurology

## Abstract

In ischemic stroke patients undergoing endovascular treatment (EVT), we aimed to test the hypothesis that cerebral microbleeds (CMBs) are associated with clinical outcomes, while estimating the mediating effects of hemorrhagic transformation (HT), small-vessel disease burden (white matter hyperintensities, WMH), and procedural success. From a multicenter EVT registry, patients who underwent pretreatment MR imaging were analyzed. They were trichotomized according to presence of CMBs (none vs. 1–4 vs. ≥ 5). The association between CMB burden and 3-month mRS was evaluated using multivariable ordinal logistic regression, and mediation analyses were conducted to estimate percent mediation. Of 577 patients, CMBs were present in 91 (15.8%); 67 (11.6%) had 1–4 CMBs, and 24 (4.2%) had ≥ 5. Increases in CMBs were associated with hemorrhagic complications (β = 0.27 [0.06–0.047], *p* = 0.010) in multivariable analysis. The CMB effect on outcome was partially mediated by post-procedural HT degree (percent mediation, 14% [0–42]), WMH (23% [7–57]) and lower rates of successful reperfusion (6% [0–25]). In conclusion, the influence of CMBs on clinical outcomes is mediated by small-vessel disease burden, post-procedural HT, and lower reperfusion rates, listed in order of percent mediation size.

## Introduction

Cerebral microbleeds (CMBs) are characterized by magnetic resonance imaging (MRI) findings of small foci of chronic blood products in the brain tissue^1^. CMBs are associated with an increased future risk of hemorrhagic and ischemic stroke^2^. They also influence acute stroke treatment, as their presence is associated with an increased risk of intracranial hemorrhage with anticoagulation/antiplatelet therapy^3^ or thrombolytic therapy^4^. Interestingly, increases in CMBs seems to be associated with a greater risk of hemorrhage, with reported increased risks with ≥ 5 CMBs^5^ or ≥ 10 CMBs^4^.

CMBs are also known to contribute to neurological dysfunction. The presence of CMBs has been associated with cognitive impairment and clinical disability^6,7^ and a significant small-vessel disease burden in the involved patients. There is a possibility that CMBs may influence endovascular treatment (EVT) outcomes by hindering a functional recovery in multiple domains following ischemic stroke^8^. Such a baseline injury to the brain may be quantitatively measured by white matter hyperintensity (WMH)^9^.

Mechanical thrombectomy for acute large-vessel occlusive stroke has become the standard of therapy^10^. It has broad efficacy over almost all subgroups, irrespective of age, sex, and stroke severity^11^. However, procedural hemorrhagic complications still substantially influence mechanical thrombectomy outcomes, and identification of predictive factors is needed to minimize and effectively manage such complications^12^. In this regard, it is of value to evaluate whether the presence of CMBs is associated with the risk of hemorrhagic complications after mechanical thrombectomy. Unfortunately, there is currently only limited literature addressing the safety of mechanical thrombectomy in patients with CMBs at presentation; moreover, meta-analyses have included only a small number of patients^13^.

For this study, we hypothesized that an increase in CMBs will negatively influence outcomes after mechanical thrombectomy, and that this effect will be mainly mediated through increases in hemorrhagic complications. We further sought to evaluate whether this negative influence was also mediated by small-vessel disease burden or other potential variables.

## Methods

### Patient enrollment and evaluations

**Figure 1:**
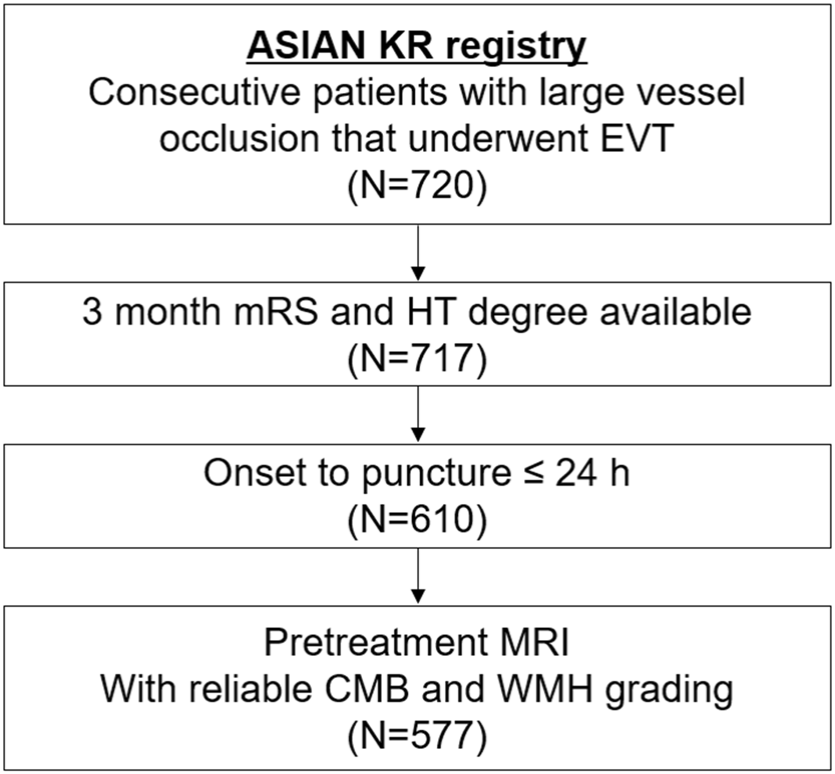
A flow chart showing the patient selection criteria for this study.

Patients were retrospectively identified from the Acute Stroke due to Intracranial Atherosclerotic occlusion and Neurointervention-Korean Retrospective (ASIAN KR) registry which included 720 consecutive patients who had undergone EVT for an emergent large-vessel occlusion between January 2011 and May 2016^14,15^. The ASIAN KR registry included patient data from three comprehensive stroke centers. All three centers used noncontrast computed tomography (CT) and CT angiography for baseline imaging. In two centers, pretreatment MRI was routinely co-performed in nearly all patients. In a third center, pretreatment MRI was usually taken in the early phase of the study period, while multiphase CT alone was used in the later phases of the study. From this registry, patients who met the following criteria were included; (1) available 3 month functional outcomes and data regarding hemorrhagic complications; (2) symptoms onset to arterial puncture time less than 24 h; and (3) pretreatment MR imaging with both T2-weighted fluid-attenuated inversion recovery and gradient-recall echo (GRE) protocols to ensure reliable CMB and WMH grading (Fig. 1). Detailed MR protocols are shown in the supplementary methods.

The 3-month modified Rankin Scale (mRS) score, which was achieved by each center through retrospective review of prospectively collected stroke registry, was utilized as clinical outcomes. After de-identification and blinding of clinical data, core laboratory imaging analysis was performed to ensure consistent grading and eliminate bias.

Regarding preprocedural diffusion-weighted images, the infarct volume was evaluated using the NordicICE semi-automated software (NordicNeuroLab, Bergen, Norway) (J.W.C., interventional neuroradiologist, 10 years of clinical experience). Successful reperfusion was defined as a modified Treatment In Cerebral Ischemia grade 2b–3^16^ (J.S.L., interventional neurologist, over 10 years of clinical experience, and Y.H.H., vascular neurologist, over 10 years of clinical experience). Hemorrhagic complications were evaluated by brain CT images in most cases or on GRE sequence MR images for CT-ineligible cases at around 5–7 days after onset. Hemorrhagic transformation (HT) degrees were graded according to the European Cooperative Acute Stroke Study II criteria^17^ (S.I.S., vascular neurologist, over 10 years of clinical experience). For statistical analysis, the grades were analyzed as ordinal; grade 0, no hemorrhage; grade 1, hemorrhagic infarct type 1 (small petechiae); grade 2, hemorrhagic infarct type 2 (more confluent petechiae); grade 3, parenchymal hematoma (PH) type 1 (≤ 30% of the infarcted area with some slight space-occupying effect); and grade 4, PH type 2 (> 30% of the infarcted area with substantial space-occupying effect).

The data collection protocol was approved by the institutional review board of each participating hospital (Ajou University Hospital IRB, Kyungpook National University Hospital IRB, and Keimyung University Dongsan Hospital IRB) and implemented under the ethical standards of the 1964 Declaration of Helsinki and its later amendments. The corresponding IRB waived the need for written informed consent due to the retrospective nature of the study.

### Identification and analysis of CMBs and WMHs

CMBs were identified in pretreatment GRE images (S.J.L., interventional neurologist, over 5 years of clinical experience) as a blooming effect. Punctate, homogenous, round/ovoid, hypointense lesions < 10 mm were identified. Previous intracranial hemorrhage was not regarded as CMBs. The exclusion of mimics was performed through the analysis of other sequences and CT imaging if needed^1^. The number of CMBs was trichotomized to: none, 1–4, and ≥ 5 according to previous literatures^18–20^.

WMHs were evaluated in the initial MR fluid-attenuated inversion recovery images or T2-weighted images according to the CREDOS WMH visual rating scale: normal, minimal, moderate, and severe ischemia (S.J.L.)^9^. Chronic territorial infarcts were not regarded as WMHs. To exclude confounding effects of acute ischemic changes to the current infarct, WMH was usually measured in the unaffected hemisphere, as there is high interhemispheric severity correlation^21^. The analysis of CMBs and WMH was performed on separate readings to ensure blinding between each other.

### Statistics

Clinical characteristics and reperfusion outcomes were compared between trichotomized CMB groups (none, 1–4 CMBs, and ≥ 5 CMBs). Univariate analysis was performed to evaluate the influence of CMBs on HT and poorer functional outcomes. For comparison of three groups, univariate analysis was performed with the χ^2^ test for categorical variables or analysis of variance with Bonferroni post-hoc tests for continuous variables. For comparison of two groups, univariate analysis was performed with the χ^2^ test for categorical variables and student’s t-test for continuous variables. The variables trichotomized CMB number, HT degree, and WMH were used as continuous variables based on trend analysis, whereas successful reperfusion and 3-month mRS was analyzed as ordinal. To identify factors associated with ordinal increases in 3-month mRS, ordinal logistic regression analysis was performed including trichotomized CMB numbers, WMH, and other clinically significant variables. To identify factors associated with increases in HT degree, multiple linear regression was performed including trichotomized CMB numbers, CMB distributions, WMH, and other clinically significant variables.

**Figure 2:**
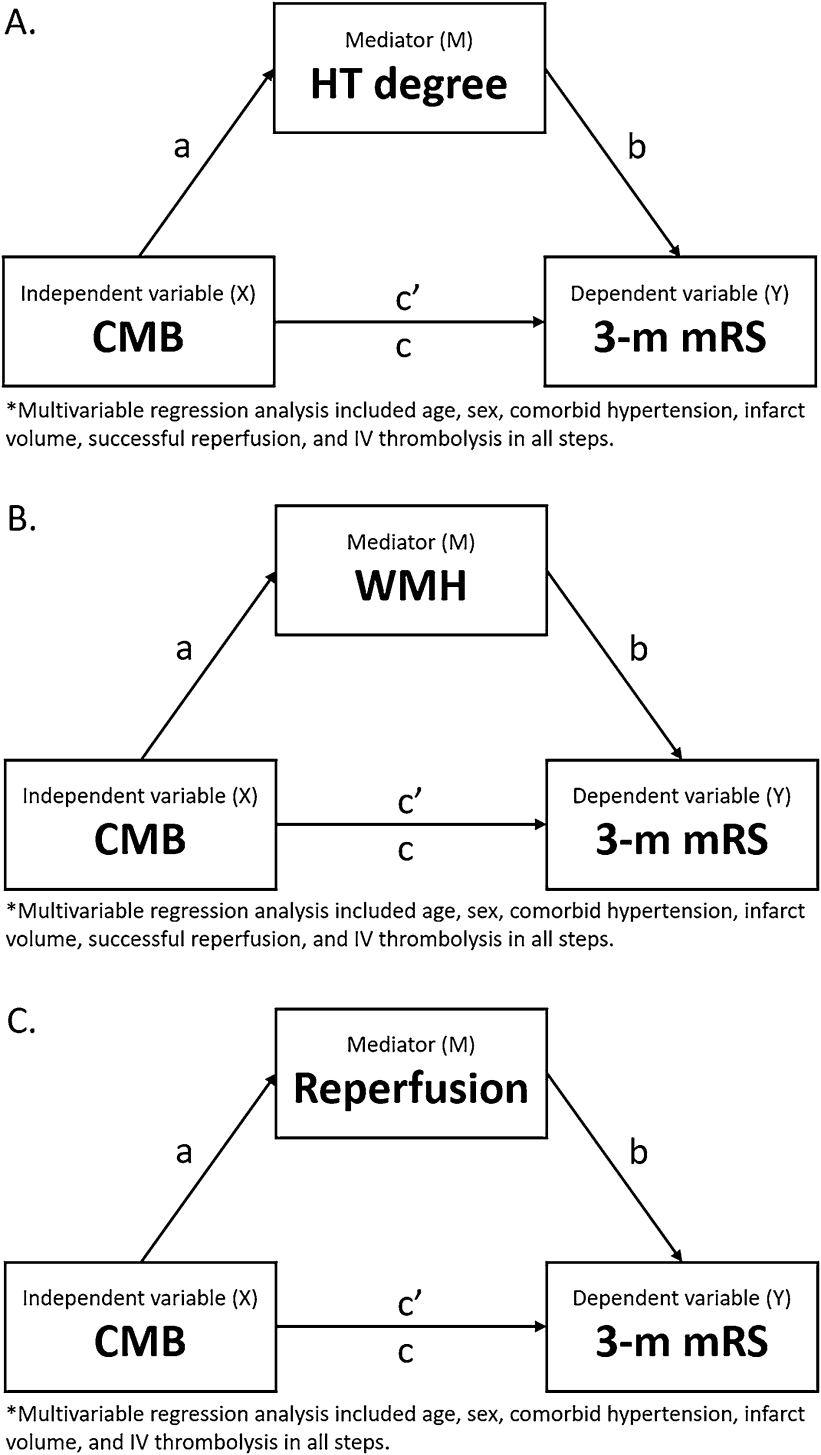
Model of the hypothetical causal pathway for the influence of CMBs on functional outcomes. (**A**) Post-procedural hemorrhagic complications measured by hemorrhagic transformation degree as a mediator. (**B**) Small-vessel disease burden (measured by white matter hyperintensities using the CREDOS criteria) as a mediator. (**C**) Successful reperfusion as a mediator.

To assess HT and WMH as potential mediators of the relationship between CMBs and poorer functional outcomes, mediation analysis was performed with the degree of HT and WMH as mediating variable, respectively. Successful reperfusion was also incorporated in the mediation analysis after univariate analyses. Mediation analysis was performed using the template described by Baron and Kenny^22^. To assess mediation, the independent variable (X) is first shown to be significantly associated with the dependent variable (Y) (Fig. 2, pathway c). Second, X must be significantly associated with the mediator (M) (Fig. 2, pathway a). Third, M is significantly associated with Y, while controlling for X (Fig. 2, pathway c’). If all the above associations are confirmed, mediation (indirect effect) can be established by estimation of the direct causal relationship.

The pathway between CMBs and mediator was tested using univariable and multivariable linear regression analyses. All other pathways were tested using univariable and multivariable ordinal regression analyses and reported as unadjusted and adjusted common odds ratio. Multivariable modeling included clinically relevant variables. The causal relationship's proportion between CMB burden and poor functional outcome attributable to the mediator was measured by dividing the log odds ratio of the indirect effect of CMB in pathway A-B by the log odds ratio of the direct effect of CMB in pathway C^23,24^. The confidence intervals for the proportion of the mediated effect were estimated using bootstrap resampling with 1000 resamples. In this approach, the 95% confidence interval can exceed 0% and 100%; however, we manually truncated the lower bound to 0% and the upper bound to 100%^25^.

Data are presented as the mean ± standard deviation, number (%), or median [interquartile range] as appropriate for data type and distribution. All statistical analyses were performed with IBM SPSS Statistics version 25 (IBM Corp., Armonk, NY) and R software, version 3.6.3. A *p* < 0.05, was considered to be statistically significant. The VGAM and boot package was used to perform the mediation analysis.

## Results

### Baseline characteristics and reperfusion outcomes

A total of 577 patients (mean age: 67 years ± 13; 322 men [55.8%]) were included in the analysis. CMB was present in 91 (15.8%) patients. Sixty-seven (11.6%) had 1–4 CMBs, whereas 24 (4.2%) had ≥ 5 CMBs. When baseline characteristics were compared between the three groups (Table 1), the mean age was higher in the CMB ≥ 5 group (66.7 ± 12.8 vs. 70.4 ± 10.6 vs. 72.0 ± 9.9, *p* = 0.01; No CMB vs. CMB ≥ 5, *p* = 0.07, post-hoc Bonferroni test), while the rates of comorbid hypertension increased with the CMB burden (288/486 [59.3%] vs. 49/67 [73.1%] vs.22/24 [91.7%], *p* = 0.001). Rates of prior stroke were higher in the presence of CMBs (78/486 [16.0%] vs. 19/67 [28.4%] vs. 5/24 [20.8%], *p* = 0.04), and WMH grades were also significantly higher with increases in the CMB burden (*p* < 0.001). However, no differences were found in the premorbid mRS (0.0 [0.0–0.0] vs. 0.0 [0.0–0.0] vs. 0.0 [0.0–0.0], *p* = 0.41).

**Table 1:** Clinical characteristics according to cerebral microbleed presence and number.

	No CMB (n = 486)	CMB 1–4 (n = 67)	CMB ≥ 5 (n = 24)	*p* value
**Clinical characteristics**				
Age, years	66.7 ± 12.8*	70.4 ± 10.6	72.0 ± 9.9*	0.01
Sex, male	274 (56.4%)	35 (52.2%)	13 (54.2%)	0.80
Premorbid mRS	0.0 [0.0–0.0]	0.0 [0.0–0.0]	0.0 [0.0–0.0]	0.41
NIHSS score	16.0 [12.0–20.0]	17.0 [12.0–21.0]	17.5 [13.25–22.00]	0.37
Glucose, mg/dL	142 ± 57	141 ± 57	138 ± 45	0.94
Infarct volume, ml	29 ± 49	30 ± 53	26 ± 42	0.94
**Occlusion location**				0.24
ICA T	158 (32.5%)	21 (31.3%)	4 (16.7%)	
MCA M1	227 (46.7%)	30 (44.8%)	17 (70.8%)	
MCA M2	39 (8.0%)	8 (11.9%)	2 (8.3%)	
VBA	54 (11.1%)	5 (7.5%)	1 (4.2%))	
Others	8 (1.6%)	3 (4.5%)	0 (0.0%)	
Diabetes mellitus	132 (27.2%)	20 (29.9%)	7 (29.2%)	0.88
Hypertension	288 (59.3%)	49 (73.1%)	22 (91.7%)	0.001
Atrial fibrillation	243 (50.0%)	33 (49.3%)	9 (37.5%)	0.49
Dyslipidemia	147 (30.2%)	23 (34.3%)	4 (16.7%)	0.27
Smoking	116 (23.9%)	15 (22.4%)	4 (16.7%)	0.70
Prior stroke	78 (16.0%)	19 (28.4%)	5 (20.8%)	0.04
**WMH**				< 0.001
None	219 (45.0%)	12 (17.9%)	2 (8.3%)	
Mild	162 (33.3%)	25 (37.3%)	5 (20.8%)	
Moderate	68 (14.0%)	17 (25.4%)	5 (20.8%)	
Severe	37 (7.6%)	13 (19.4%)	12 (50.0%)	

The data are presented as the mean ± standard deviation, number (%), or median [interquartile range], as appropriate.

*CMB* cerebral microbleeds, *ICA* internal carotid artery, *MCA* middle cerebral artery, *NIHSS* National Institutes of Health Stroke Scale, *VBA* vertebrobasilar artery, *WMH* white matter hyperintensity.

*No CMB versus CMB ≥ 5, *p* = 0.07, post-hoc Bonferroni test.

**Figure 3:**
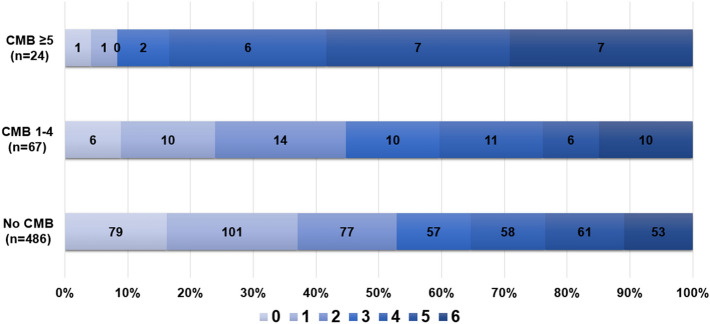
The ordinal distribution of 3 month mRS according to CMB burden. *mRS* modified Rankin Scale, CMB, *cerebral microbleeds*.

Regarding procedural characteristics and reperfusion outcomes (Table 2), significant differences were observed in the rates of reperfusion success, with lower rates of successful reperfusion in the CMB ≥ 5 group (382/486 [78.6%] vs. 54/67 [80.6%] vs. 13/24 [54.2%], *p* = 0.02). While the overall rate of difference in HT was not significant between groups, there was a significant intergroup difference in PH rates (59/486 [12.1%] vs. 9/67 [13.4%] vs. 8/24 [33.3%], *p* = 0.01), especially for the CMB ≥ 5 group. Median 3-month mRS was significantly higher in the CMB ≥ 5 group (2.0 [1.0–4.0] vs. 3.0 [2.0–4.0] vs. 5.0 [4.0–6.0], *p* < 0.001; No CMB and CMB 0–4 vs. CMB ≥ 5, *p* < 0.005, post-hoc Bonferroni test). An ordinal distribution of the 3 month mRS is shown for each CMB group in Fig. 3.

**Table 2:** Procedural characteristics, imaging, and clinical outcomes according to the number and presence of cerebral microbleeds.

	No CMB (n = 486)	CMB 1–4 (n = 67)	CMB ≥ 5 (n = 24)	*p* value
Onset to door, min	228 ± 215	257 ± 231	274 ± 212	0.38
Onset to reperfusion, min	421 ± 244	464 ± 244	473 ± 211	0.26
IV thrombolysis	254 (52.3%)	33 (49.3%)	13 (54.2%)	0.88
**Primary EVT modality**				0.30
Stent retrieval	153 (31.5%)	18 (26.9%)	10 (41.7%)	
Direct aspiration	309 (63.6%)	42 (62.7%)	13 (54.2%)	
Others	24 (4.9%)	7 (10.4%)	1 (4.2%)	
Balloon guide catheter	308 (63.4%)	45 (67.2%)	14 (58.3%)	0.72
Successful reperfusion	382 (78.6%)	54 (80.6%)	13 (54.2%)	0.02
Final infarct volume, ml	57 ± 70 (n = 387)	51 ± 73 (n = 53)	72 ± 82 (n = 16)	0.57
**HT grade**				0.07
No	348 (71.6%)	47 (70.1%)	12 (50.0%)	
HI 1	31 (6.4%)	5 (7.5%)	2 (8.3%)	
HI 2	48 (9.9%)	6 (9.0%)	2 (8.3%)	
PH1	31 (6.4%)	4 (6.0%)	2 (8.3%)	
PH2	28 (5.8%)	5 (7.5%)	6 (25.0%)	
PH	59 (12.1%)	9 (13.4%)	8 (33.3%)	0.01
SAH grade 3–4	20 (4.1%)	1 (1.5%)	0 (0.0%)	0.35
3 month mRS	2.0 [1.0–4.0]	3.0 [2.0–4.0]	5.0 [4.0–6.0]	< 0.001*

The data are presented as the mean ± standard deviation, number (%), or median [interquartile range], as appropriate.

*CMB* cerebral microbleeds, *EVT* endovascular treatment, *HI* hemorrhagic infarct, *HT* hemorrhagic transformation, *IV* intravenous, *mRS* modified Rankin Scale, *NIHSS* National Institutes of Health Stroke Scale, *PH* parenchymal hematoma, *VBA* vertebrobasilar artery, *WMH* white matter hyperintensity.

*No CMB and CMB 0–4 versus CMB ≥ 5, *p* < 0.005, post-hoc Bonferroni test.

### Factors associated increases in 3 month mRS and HT degree

In the multivariable analysis identifying factors associated with increases in 3 month mRS, increases in trichotomized CMB numbers (OR: 1.42 [95% CI 1.04–1.94], *p* = 0.028), increases in HT grades (OR: 1.48 [95% CI 1.31–1.68], *p* < 0.001), and increases in WMH severity (OR: 1.27 [95% CI 1.06–1.53], *p* = 0.01) were associated with increases in 3 month mRS with successful reperfusion, age, sex, comorbid hypertension, infarct volume, and premorbid mRS as co-variables (Table 3).

**Table 3:** Multivariable analysis identifying predictors of 3 month modified Rankin Scale scores as ordinal outcome.

	OR (95% CI)	*[* value
Trichotomized CMB numbers	1.42 [1.04–1.94]	0.028
WMH	1.27 [1.06–1.53]	0.01
HT grades	1.48 [1.31–1.68]	< 0.001
Age	1.02 [1.01–1.04]	< 0.001
Sex, male	1.00 [0.74–1.36]	0.99
Comorbid hypertension	1.15 [0.83–1.58)	0.41
Infarct volume	1.01 [1.01–1.02]	< 0.001
Successful reperfusion	0.33 [0.23–0.48]	< 0.001
IV thrombolysis	0.74 [0.55–1.00]	0.048
**Premorbid_mRS**		
0	Ref	
1	1.55 [0.94–2.53]	0.08
2	1.74 [0.9–3.37]	0.10
3	4.71 [2.16–10.25]	< 0.001
4	5.57 [1.61–19.25]	0.007

*CMB* cerebral microbleeds, *HT* hemorrhagic transformation, *IV* intravenous, *mRS* modified Rankin Scale, *WMH* white matter hyperintensity.

In the multivariable analysis identifying factors associated with increases in HT, increases in trichotomized CMB numbers (β = 1.00 [95% CI 0.39–1.61], *p* = 0.001) were associated with increases in HT, while WMH (β = − 0.1 [95% CI − 0.22 to 0.02], *p* = 0.108), and IV thrombolysis (β = − 0.14 [95% CI − 0.34 to 0.06], *p* = 0.163) was not, along with age, sex, comorbid hypertension, infarct volume, and successful reperfusion as co-variables (Table 4).

**Table 4:** Multivariable analysis identifying predictors of HT grade as a continuous variable.

	β (95% CI)	*p* value
Trichotomized CMB numbers	1.00 [0.39–1.61]	0.001
WMH	− 0.1 [− 0.22 to 0.02]	0.108
IV thrombolysis	− 0.14 [− 0.34 to 0.06]	0.163
Age	0.01 [0–0.02]	0.203
Sex, male	− 0.14 [0.34–0.06]	0.178
Comorbid hypertension	0.00 [− 0.22 to 0.21]	0.981
Infarct volume	0.01 [0.01–0.01]	< 0.001
Successful reperfusion	− 0.05 [− 0.29 to 0.19]	0.686

*CMB* cerebral microbleeds, *HT* hemorrhagic transformation, *IV* intravenous, *mRS* modified Rankin Scale, *WMH* white matter hyperintensity.

### Mediation by HT concerning the association between CMBs and poorer functional outcomes

In the first step of the mediation analysis, CMBs were significantly predictive of a higher 3-month mRS with an adjusted common odds ratio (acOR) of 1.77 [95% CI 1.31–2.38]. In the second step, CMBs were significantly associated with HT degree with a beta of 0.27 [95% CI 0.06–0.47]. In the third step, the direct effect of CMBs on poorer functional outcome remained statistically significant after adjustment for HT degree with an acOR 1.63 [95% CI 1.21–2.20]. The mediator HT was an independent variable and significantly associated with poorer functional outcomes with an acOR of 1.43 [95% CI 1.26–1.62]. HT was attributed to 14% [95% CI 0–42] of the total influence of CMBs on functional outcomes (Table 5). Age, sex, comorbid hypertension, infarct volume, successful reperfusion, and IV thrombolysis were included as covariates in all multivariable analyses. In the analysis of CMB influence on HT degree, there was no collinearity effect between CMB and successful reperfusion (VIF for CMB: 1.04, VIF for successful reperfusion: 1.03).

**Table 5:** Association of trichotomized CMB numbers with functional outcomes mediated by hemorrhagic transformation degree, white matter hyperintensities, and reperfusion success.

Pathway	Unadjusted			Adjusted*		
	Parameter	Value (95% CI)	*p* value	Parameter	Value (95% CI)	*p* value
**Hemorrhagic transformation**						
a (CMB → HT)	β	0.27 [0.06–0.48]	0.011	β	0.27 [0.06–0.047]	0.010
b (HT → mRS)	cOR	1.63 [1.44–1.83]	< 0.001	acOR	1.46 [1.29–1.65])	< 0.001
c (CMB → mRS)	cOR	1.88 [1.41–2.49]	< 0.001	acOR	1.77 [1.31–2.38]	< 0.001
c' (CMB + HT → mRS)	cOR	1.70 [1.27–2.28]	< 0.001	acOR	1.63 [1.21–2.20]	0.001
ß mediator	cOR	1.60 [1.42–1.80]	< 0.001	acOR	1.43 [1.26–1.62]	< 0.001
Percent mediation	%	15 [0–41]		%	14 [0–42]	
**WMH**						
a (CMB → WMH)	β	0.63 [0.48–0.79]	< 0.001	β	0.50 [0.36–0.64]	< 0.001
b (WMH → mRS)	cOR	1.58 [1.37–1.83]	< 0.001	acOR	1.41 [1.19–1.67]	< 0.001
c (CMB → mRS)	cOR	1.88 [1.41–2.49]	< 0.001	acOR	1.77 (1.31–2.38)	< 0.001
c' (CMB + WMH → mRS)	cOR	1.49 [1.10–2.01]	0.009	acOR	1.55 [1.13–2.11]	0.006
ß mediator	cOR	1.49 [1.28–1.74]	< 0.001	acOR	1.31 [1.10–1.57]	0.003
Percent mediation	%	37 [19–74]		%	23 [7–57]	
**Successful reperfusion**				†		
a (CMB → reperfusion)	cOR	0.70 [0.49–1.01]	0.055	acOR	0.67 [0.46–0.97]	0.034
b (reperfusion → mRS)	cOR	0.32 [0.22–0.45]	< 0.001	acOR	0.33 [0.23–0.47]	< 0.001
c (CMB → mRS)	cOR	1.88 [1.41–2.49]	< 0.001	acOR	1.84 [1.37–2.47]	< 0.001
c' (CMB + reperfusion → mRS)	cOR	1.82 [1.37–2.42]	< 0.001	acOR	1.77 [1.31–2.38]	< 0.001
ß mediator	cOR	0.32 [0.23–0.46]	< 0.001	acOR	0.34 [0.24–0.49]	< 0.001
Percent mediation	%	5 [0–21]		%	6 [0–25]	

β mediator: coefficient of mediator in pathway X + M → Y.

*acOR* adjusted common odds ratio, *cOR* common odds ratio, *CI* confidence interval, *CMB* cerebral microbleeds, *mRS* modified Rankin Scale, *WMH* white matter hyperintensity.

*Adjusted for age, sex, comorbid hypertension, infarct volume, successful reperfusion, and IV thrombolysis.

^†^Adjusted for age, sex, comorbid hypertension, infarct volume, and IV thrombolysis.

In the eight patients with ≥ 5 CMBs and a post-procedural PH, hemorrhage occurred at the anatomical CMBs location in three cases (37.5%), whereas in five cases (63.5%), PH occurred at an anatomically irrelevant area.

### Mediation by WMH concerning the association between CMBs and poorer functional outcomes

In the first step of mediation analysis, CMBs were significantly predictive of higher 3-month mRS with an acOR of 1.77 [95% CI 1.31–2.38]. In the second step, CMBs were significantly associated with WMH with a beta of 0.50 [95% CI 0.36–0.64]. In the third step, the direct effect of CMBs on poorer functional outcome remained statistically significant after adjustment for WMH with an acOR 1.55 [95% CI 1.13–2.11]. The mediator WMH was an independent variable and significantly associated with poorer functional outcomes with an acOR of 1.31 [95% CI 1.10–1.57]. WMH was attributed to 23% [95% CI 7–57] of the total influence of CMBs on functional outcomes (Table 5). Age, sex, comorbid hypertension, infarct volume, successful reperfusion, and IV thrombolysis were included as covariates in all multivariable analyses.

### Mediation by successful reperfusion concerning the association between CMBs and poorer functional outcomes

As the lower rate of successful reperfusion was an unexpected finding for groups with higher CMB burden, further mediation analysis was performed with successful reperfusion as mediator. In the first step of mediation analysis, CMBs were significantly predictive of higher 3-month mRS with an acOR of 1.84 [95% CI 1.37–2.47]. In the second step, CMBs showed a significant inverse correlation with successful reperfusion with an acOR of 0.67 [95% CI 0.46–0.97]. In the third step, the direct effect of CMBs on poorer functional outcome remained statistically significant after adjustment for successful reperfusion with an acOR 1.77 [95% CI 1.31–2.38]. The mediator successful reperfusion was an independent variable and significantly associated with poorer functional outcomes with an acOR of 0.34 [95% CI 0.24–0.49]. Lower rates of successful reperfusion were attributed to 6% [95% CI 0–25] of the total influence of CMBs on poorer functional outcomes (Table 5). Age, sex, comorbid hypertension, infarct volume, and IV thrombolysis were included as covariates in all multivariable analyses.

## Discussion

This study shows that increases in CMB burden are associated with worsened functional outcomes after endovascular treatment, and reveals the factors that act as mediators of outcomes. As per previous concerns, CMB burden was associated with an increased incidence of post-procedural hemorrhagic complications, resulting in worse functional outcomes. However, hemorrhagic complications were only partly accountable for the poorer functional outcomes. The influence of a high CMB burden on outcomes was partially mediated by white matter hyperintensity at a greater degree, while lower rates of successful reperfusion played a smaller role.

The current study revealed that the rate of hemorrhagic complications significantly increased with the CMB burden, independent of other covariates. Previous reports regarding this issue are somewhat controversial. However, advances in endovascular treatment methods and MR imaging methods may be the cause of the different results. Results of a recent meta-analysis involving 598 patients regarding the association between post-mechanical thrombectomy hemorrhagic complications and CMBs^13^ did not show a positive association. When we take a closer look into the studies analyzed, a previous study including Asian patients from 2002 to 2012 using 1.5T MRI GRE failed to show an association between CMB burden and hemorrhagic complications. However, in the study, only a small number of patients with ≥ 5 CMBs were included, and Merci retrievers were used in most cases^26^. Another study including European patients from 2010 to 2013 used a 1.5-T MRI and susceptibility weighted imaging. They reported the percentage of patients with ≥ 5 CMBs to be 2.3% (9/392) and a marginally increased risk for CMB to be associated with the risk for ICH^27^. Another study, which was not included in the meta-analysis, enrolled 1532 patients treated with intravenous thrombolysis or mechanical thrombectomy using modern devices between 2007 and 2017^19^. Contrary to the total population, in the 595 patients with recanalization, CMBs—especially with a high burden and lobar location—were independently associated with poor 3-month clinical outcomes and risk of symptomatic intracranial hemorrhage. Considering that our study used 3T MRI and included patients between 2011 and 2016, it is likely that more sensitive quantification of CMB burden and more consistent reperfusion with modern thrombectomy devices may result in the positive associations between CMB burden and hemorrhagic complications.

Functional outcomes were also negatively influenced by CMBs presence significantly, while this association was only partially mediated by hemorrhagic complications. CMBs are known to be markers of small-vessel disease burden, which may influence outcomes^28^. Thus, CMB influence may be potentially mediated by pathways other than hemorrhagic complications. If this is the case, CMB degree may be associated with stroke outcomes overall, and not just thrombolysis or EVT populations. However, data regarding functional outcomes are scarce, and only recently, was association between CMB burden and functional outcomes shown in a group of minor ischemic stroke patients^29^. To clarify this interrelation, we used mediation analysis to explain the influence of CMBs on outcomes mediated by both HT and WMH (small-vessel disease burden); showing that both factors mediated the negative influence of CMBs on outcomes. While both CMBs and WMHs are considered as imaging biomarkers of small-vessel disease, WMHs were used as a proxy for overall small-vessel disease burden in this study to represent the chronic brain injury associated with the small-vessel disease, and also because WMHs are relatively frequent, with well-validated grading systems. There is increasing evidence that WMH represents macroscopic injury to the white matter, and its extent influences functional recovery in multiple domains following ischemic stroke^8^. Further, WMH predicts post-stroke cognitive performance after stroke well among small-vessel disease markers^30^. Clinically, CMBs and WMHs share common risk factors, such as hypertension and diabetes. Our results remained statistically significant even after controlling for such factors.

An unexpected result of our study is the lower rate of reperfusion with increasing CMB burden. Due to retrospective imaging analysis, cause of reperfusion failure could not be clearly identified in this study. Recent studies have reported associations between small vessel disease markers and intracranial arterial dolichoectasia^31^. Such increases in the arterial tortuosity may theoretically interfere with lesion access, device delivery, and clot retrieval^32^. However, the lower reperfusion rates accounted for 6% of the total effect of CMB burden on functional outcomes, which was lower than that of other variables. Furthermore, the CMB effect on outcomes mediated by post-procedural HT degree was independent of successful reperfusion as per our hypothesis.

The study results must be interpreted with some caution. First, although poor outcomes were observed in the high CMB burden group, this effect was not purely due to increases in hemorrhagic complications. The results of this study do not suggest absence of treatment effect of EVT in the high CMB population, and should not be used as evidence for excluding patients from a powerful treatment modality. A recent study using data from a randomized IV thrombolysis has shown no reduced treatment effect of alteplase in acute ischemic stroke patients with one or more CMBs^33^, while reaching inconclusive conclusions regarding larger numbers of CMBs. Similarly designed studies will be needed regarding EVT treatment effect. In the time being, our results support the cumulative evidence of CMBs as a risk factor of HT, establishing CMB as an imaging biomarker that deserves attention. Second, there were differences in baseline characteristics according to increasing CMB count, such as increased age or reperfusion rates; however, after correcting for these factors, our results showed that both the influence on outcomes and the mediation effect remained statistically significant. Third, a simple 4-point visual scale was used to grade WMH, which might attenuate its effect. There is a chance that when WMH is quantitatively analyzed^34^, the percent mediation may increase. Third, current treatment recommendations advocate a reduction of door-to-puncture time^35^, and the use of advanced imaging for routine patient selection may be debatable. Although the most appropriate imaging method requires further evidence, screening for high CMB burden may be an advantage of MR-based imaging.

## Summary

In conclusion, a high CMB burden was associated with poorer outcomes in stroke patients undergoing mechanical thrombectomy. Our results show that this can be explained by higher small vessel disease burden that may hinder neurological recovery, increases in post-procedural HT complications, and lower reperfusion rates, listed in order of percent mediation size.

## Supplementary Information

Supplementary Information.

## Data Availability

The datasets generated during the current study are available from the corresponding author on reasonable request.

## Abbreviations

acOR: Adjusted common odds ratio
CMB: Cerebral microbleed
CI: Confidence interval
EVT: Endovascular treatment
GRE: Gradient echo protocol
HT: Hemorrhagic transformation
mRS: Modified Rankin Scale
PH: Parenchymal hematoma
WMH: White matter hyperintensity
