# Supplementary material for: Influence of cerebral microbleeds on mechanical thrombectomy outcomes
Source: Sci Rep. 2022 Mar 7;12:3637. doi: 10.1038/s41598-022-07432-9 (PMC8901625; doi:10.1038/s41598-022-07432-9)
Supplement: Supplementary file 1 — Supplementary Information. [file 41598_2022_7432_MOESM1_ESM.docx]

**Influence of cerebral microbleeds on mechanical thrombectomy outcomes**

**Short title:** CMB influence on thrombectomy outcomes

Seong-Joon Lee, MD, PhD^1^,* Yang-Ha Hwang, MD, PhD^2^,* Ji Man Hong, MD, PhD^1^, Jin Wook Choi, MD, PhD^3^, Ji Hyun Park, MS^4^, Bumhee Park, PhD^4,5^, Dong-Hun Kang, MD^6,7^, Yong-Won Kim, MD^2,7^, Yong-Sun Kim MD, PhD^7^, Jeong-Ho Hong, MD, PhD^8^, Joonsang Yoo, MD^8,9^, Chang-Hyun Kim, MD^10^, Sung-Il Sohn, MD, PhD^8^, Jin Soo Lee, MD, PhD^1^

^1^Department of Neurology, Ajou University School of Medicine, Ajou University Medical Center, Suwon, Republic of Korea

^2^Department of Neurology, School of Medicine, Kyungpook National University, Daegu, Republic of Korea

^3^Department of Radiology, Ajou University School of Medicine, Ajou University Medical Center, Suwon, Republic of Korea

^4^Office of Biostatistics, Medical Research Collaborating Center, Ajou Research Institute for Innovative Medicine, Ajou University Medical Center, Suwon, Republic of Korea

^5^Department of Biomedical Informatics, Ajou University School of Medicine, Suwon, Republic of Korea

^6^Department of Neurosurgery, School of Medicine, Kyungpook National University, Daegu, Republic of Korea

^7^Department of Radiology, School of Medicine, Kyungpook National University, Daegu, Republic of Korea

^8^Department of Neurology, Keimyung University Dongsan Medical Center, Daegu, Republic of Korea

^9^ Department of Neurology, National Health Insurance Service Ilsan Hospital, Goyang, Korea

^10^Department of Neurosurgery, Keimyung University Dongsan Medical Center, Daegu, Republic of Korea

*These authors contributed equally to the manuscript.

**Corresponding author:**

Jin Soo Lee, MD, PhD

Department of Neurology, Ajou University School of Medicine

164, World cup-ro, Yeongtong-gu, Suwon, Gyeonggi-do, 16499, South Korea

Phone: +82-31-219-5175

Fax: +82-31-219-5178

E-mail: jinsoo22@gmail.com

**Keywords**: cerebral microbleed, mechanical thrombectomy, white matter disease, intracranial hemorrhage

**Manuscript Type:** Original Article

**SUPPLEMENTARY MATERIALS**

Supplementary Methods

For center A, brain MRI was performed with Achieva 3.0 T (Philips Healthcare, Amsterdam, Netherlands) or Discovery^TM^ MR750W 3.0 T (GE Medical Systems, Milwaukee, Wis). A 32-channel head coil was used. Protocol for DWI was, TR/TE: 3668/69 ms; FA: 90; FOV: 220 × 220; NEX: 2; slice thickness: 5 mm; interslice gap: 0 mm; matrix size: 160 × 160; b: 1000 sec/mm^2^. Protocol for GRE images was, TR/TE/: 695/16 ms; FA: 20; FOV: 200 × 200; NEX: 1; slice thickness: 5mm; interslice gap: 0 mm; matrix size: 240 × 176. Protocol for FLAIR images was, TR/TE: 11000/125 ms; TI: 2400 ~ 2800 ms; FA: 160; FOV: 200 × 200; NEX: 1; slice thickness: 5mm; interslice gap: 0 mm; matrix size: 300 × 176.

For center B, Brain MRI was performed with Discovery^TM^ MR750W 3.0 T (GE Medical Systems, Milwaukee, Wis). A 32-channel head coil was used. Protocol for DWI was, TR/TE: 10000/75 ms; FA: 90; FOV: 260 × 260; NEX: 2; slice thickness: 4 mm; interslice gap: 0.4 mm; matrix size: 192 × 160; b: 1000 sec/mm^2^. Protocol for GRE images was, TR/TE: 517/18 ms; FA: 15; FOV: 220 × 220; NEX: 1; slice thickness: 5mm; interslice gap: 2 mm; matrix size: 320 × 224. Protocol for FLAIR images was, TR/TE: 8000/145 ms; TI: 1961 ms; FA: 142; FOV: 220 × 220; NEX: 1; slice thickness: 5mm; interslice gap: 2 mm; matrix size: 384 × 224.

For center C, brain MRI was performed with SIGNA^TM^ 3.0T (GE Medical Systems, Milwaukee, Wis). An 8-channel head coil was used. Protocol for DWI was, TR/TE: 6400/817 ms; FA: 90; FOV: 230 × 230; NEX: 1; slice thickness: 5mm; interslice gap: 0 mm; matrix size: 160 × 160; b: 0, 1000 sec/mm^2^. Protocol for GRE images was, TR/TE: 500/20 ms; FA: 20; FOV: 300 × 300; NEX: 1; slice thickness: 5mm; interslice gap: 0.4 mm; matrix size: 512 × 160. Protocol for FLAIR images was, TR/TE: 8000/138.1 ms; TI: 2370; FA: 90; FOV: 230 × 230; NEX: 1; slice thickness: 5mm; interslice gap: 0.4 mm; matrix size: 256 × 150.
